# Association of hyperkalaemia with electrocardiographic changes at a tertiary centre in South Africa

**DOI:** 10.4102/jcmsa.v3i1.110

**Published:** 2025-04-30

**Authors:** Francis R. Ssenabulya, Mogamat-Yazied Chothia

**Affiliations:** 1 Division of Nephrology, Department of Medicine, Faculty of Medicine and Health Sciences, Stellenbosch University, Cape Town, South Africa

**Keywords:** hyperkalaemia, ECG, predictors, Africa, correlation

## Abstract

**Background:**

Hyperkalaemia is a common electrolyte disorder in hospitalised patients and is associated with fatal cardiac arrhythmias. In sub-Saharan Africa, there is a paucity of data regarding the prevalence and type of electrocardiographic (ECG) changes in patients who have hyperkalaemia.

**Methods:**

A retrospective descriptive study was conducted at Tygerberg Hospital over a one-year period in 2019. Adult patients with hyperkalaemia of ≥ 5.5 mmol/L and associated ECG changes 3 h before or after documented laboratory hyperkalaemia were included. Spearman correlation coefficients and multilinear regression were used to identify correlations and associations, respectively, between serum potassium concentrations ([K^+^]) and various ECG changes.

**Results:**

Of 344 patients who had hyperkalaemia and an associated ECG, 55% had ECG changes. These patients were older (60 years vs. 53 years, *p* = 0.01), male (57% vs. 43%, *p* < 0.01) and had more frequent kidney disease (88% vs. 78%, *p* = 0.02). Statistically significant differences in all ECG measurements were present, except for *T* wave amplitude. The most frequent ECG alterations were *p*-wave abnormalities (52%) and peaked *T* waves (45%). A weak-to-moderate correlation was present for the number of ECG changes and the [K^+^]. The QRS duration (*β*: 0.0076, *p* < 0.001), PR interval (*β*: 0.0039, *p* < 0.001) and *p* wave duration (*β*: –0.0056, *p* < 0.01) were associated with the [K^+^].

**Conclusion:**

The overall prevalence of ECG changes due to hyperkalaemia was only 55%.

**Contribution:**

It is essential for clinicians to recognise that the ECG changes during hyperkalaemia may have limited screening value.

## Introduction

Hyperkalaemia is a common electrolyte disorder in hospitalised patients and is associated with high in-hospital all-cause mortality.^1,2^ A systematic review and meta-analysis reported a prevalence of hyperkalaemia among adult patients of 6.3%, while the incidence of hyperkalaemia in the adult population was 2.8 cases per 100 person-years.^3^ In South Africa, the largest retrospective cohort study on hyperkalaemia in hospitalised adults reported an incidence rate of 3.7 cases per 100 patient-years.^2^

Among patients with hyperkalaemia, symptoms are often absent or mild. The electrocardiogram (ECG) is a point-of-care tool that can assist in screening as there is an association between the severity of hyperkalaemia and the evolution of ECG manifestations.^4^ The ECG plays a key role in managing hyperkalaemia by (1) screening for potential signs of the condition, which are then confirmed by laboratory testing; (2) guiding emergency treatment in cases of life-threatening hyperkalaemia, particularly when characteristic ECG patterns are present, often prompting immediate intervention without waiting for confirmatory blood tests and (3) assessing patients with known hyperkalaemia to determine who requires potassium-shifting treatment.

In South Africa, in a survey conducted regarding the knowledge of medical specialists about the emergency management of hyperkalaemia, three-quarters of respondents thought that there was a poor correlation between [K^+^] and the presence of ECG changes.^5^ There are many factors that lead to this disconnection between evolution of ECG changes and levels of hyperkalaemia that are often overlooked. These include physiologic adaptation, structural heart disease, drugs and severity of concurrent illness.^6^

A retrospective study reported that all patients with severe hyperkalaemia who developed short-term adverse events had a preceding ECG alteration. Changes associated with short-term adverse events were QRS prolongation, bradycardia and/or junctional rhythm. There was no statistically significant correlation between peaked *T* waves and short-term adverse outcomes.^7^

In our setting, hyperkalaemia occurs more frequently in younger individuals compared to those in high-income countries.^2^ The causes of hyperkalaemia related to acute kidney injury (AKI) can differ from those in developed nations, including infectious diseases such as malaria, a higher incidence of trauma-related AKI and the more common use of medications like trimethoprim in individuals living with human immunodeficiency virus (HIV). Additionally, due to limited access to healthcare, patients often present later, which can result in more severe hyperkalaemia. We could not identify any studies that investigated the prevalence and type of ECG changes associated with hyperkalaemia from sub-Saharan Africa. Therefore, we investigated this knowledge gap.

## Methods

We performed a retrospective descriptive study at Tygerberg Hospital (TBH) located in Cape Town, South Africa, involving all adult patients with hyperkalaemia over a 1-year period from 01 January 2019 to 31 December 2019. Tygerberg Hospital has 1380 beds and provides services to approximately 2.5 million people.

These were admitted patients on all adult wards, including all emergency departments, internal medicine, all surgical disciplines including general surgery, vascular, cardiothoracic, neurosurgery, obstetrics and gynaecology, ear nose and throat, ophthalmology and orthopaedics, identified with the assistance from the National Health Laboratory Service (NHLS), the national reference laboratory. All patients older than 18 years with a laboratory [K^+^] *≥* 5.5 mmol/L and an ECG performed within a window of 3 h before or after documented laboratory hyperkalaemia were included.

**FIGURE 1:**
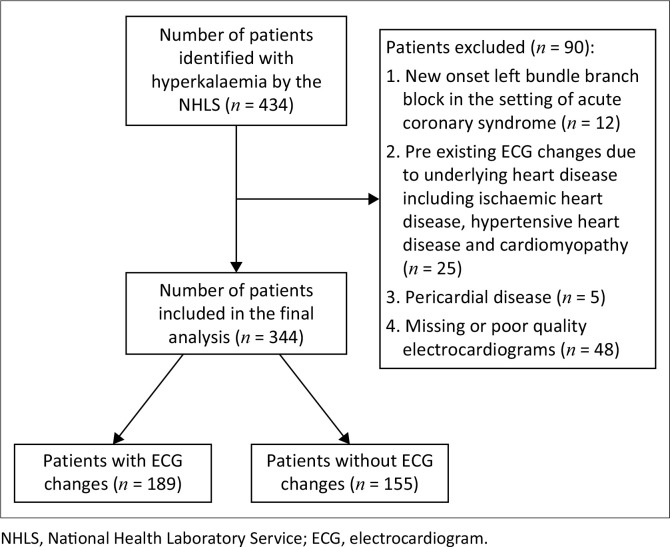
Consort diagram.

Exclusion criteria involved patients with; (1) laboratory documentation of haemolysed laboratory blood specimens, (2) outpatients, (3) patients with ECG changes prior to documentation of hyperkalaemia including pre-existing bundle branch blocks, hypertensive heart disease, ischaemic heart disease or pericardial disease, (4) persistence of ECG changes despite correction of hyperkalaemia, (5) pacemaker rhythm, (6) ECG that were uninterpretable because of poor quality and (7) no ECG identified (Figure 1).

Data extracted from electronic patient medical records included demographic and clinical characteristics like age, sex, comorbid diseases including diabetes mellitus, hypertension and heart disease, HIV status, kidney disease (AKI and chronic kidney disease [CKD]) as documented by clinicians within the medical records, drug prescriptions associated with hyperkalaemia and in-hospital outcome. Laboratory data included the serum potassium concentration [K^+^], arterial or venous blood gas for total and ionised calcium concentration and acid-base parameters, serum creatine phosphokinase level and serum creatinine concentration. Comparisons regarding demographic, clinical and laboratory data were performed between patients who did and did not have ECG changes.

Electrocardiograms were included when the calibration was 10 mm or 1 mV and the speed was 25 mm per second. All ECGs were analysed independently by two investigators (FRS and MYC). Electrocardiograms were analysed for the presence or absence of changes caused by hyperkalaemia. These included small or flat or absent *p* waves, prolonged PR interval, widened QRS complex, corrected QT interval (Hodges) and peaked *T* waves (measured amplitude in mm). Except for *T* wave amplitude, all other measurements were derived from ECG-generated intervals. Comparisons in these measurements were made between patients who did and did not have ECG changes. Conflicts were resolved through discussion and reaching consensus. Table 1 shows the criteria used to define the ECG changes ascribed to hyperkalaemia.

**TABLE 1:** Criteria used to define the electrocardiographic changes ascribed to hyperkalaemia.

Electrocardiogram segment	Definition
*T* waves	*T* waves were considered peaked when the tallest *T* wave amplitude was > 8 mm and limbs were symmetrical.
Corrected QT interval (QTc)	The Hodges formula was used to calculate corrected QT interval and was corrected for heart rate. A prolonged QTc was defined as > 450 ms in men and 470 ms in women.^8^
*P* wave amplitude and duration	We documented whether *p* waves were absent or flat (< 2.5 mm), and prolonged *p* wave was defined as > 120 ms.^9^
PR interval	Prolonged PR interval was defined as > 200 ms.^9^
QRS complex	Wide QRS complex was defined as > 120 ms.^9^

Note: Please see the full reference list of the article, Ssenabulya FR, Chothia M-Y. Association of hyperkalaemia with electrocardiographic changes at a tertiary centre in South Africa. J Coll Med S Afr. 2025;3(1), a110. https://doi.org/10.4102/jcmsa.v3i1.110, for more information.

### Ethical considerations

Ethical clearance to conduct this study was obtained from the Stellenbosch University Health Research Ethics Committee 2 (HREC2)(No. S19/05/092). A waiver of informed consent was granted due to the retrospective study design. The study was conducted in accordance with the Declaration of Helsinki.

### Statistical analysis

Statistical analyses were performed using STATA IC version 16.1, Stata Corp LLC, Texas, USA. Descriptive continuous data with a normal distribution were described using means and standard deviations, while non-normal data were reported as median and interquartile ranges. Bar graphs were used where appropriate. Chi-squared or Fisher’s exact test was used to identify statistical significance for all categorical variables. The student *t*-test was used to compare the means of continuous variables where the data had a normal distribution. Where continuous variables were not normally distributed, the Mann-Whitney *U* test was used. Spearman correlation coefficients were used to identify the strength of correlation between [K^+^] and various ECG changes. Multilinear regression was performed to identify which ECG changes were associated with the [K^+^]. The variance inflation factor was used to identify the presence of multicollinearity. Statistical significance was set at a *p*-value < 0.05 and 95% confidence intervals were used.

## Results

A total of 434 patients were identified with hyperkalaemia, of whom 344 met inclusion criteria (Figure 1). Of 344 patients found to have hyperkalaemia and an ECG performed, 189 (55%) had ECG changes. More patients who had ECG changes were men (57% vs. 43%, *p* < 0.01), were older age (60 years vs. 53 years, *p* = 0.01) and had kidney disease (88% vs. 78%, *p* = 0.02), although there were no differences regarding AKI or CKD between groups. Angiotensin-converting enzyme inhibitors were the most frequently prescribed drug associated with hyperkalaemia (Table 2).

**TABLE 2:** Baseline characteristics.

Demography, laboratory tests and in-hospital mortality	ECG changes						*p*-value
	Yes (*N* = 189; 55%)			No (*N* = 155; 45%)			
	*n*	%	Median (IQR)	*n*	%	Median (IQR)	
**Demographic characteristics**							
Age (years)	60	-	44–68	53	-	42–65	0.01
Sex (male)	108	57.0	-	66	43.0	-	< 0.01
**Comorbidities**							
HIV positive	22	12.0	-	20	13.0	-	0.54
Hypertension	121	64.0	-	85	55.0	-	0.08
Diabetes mellitus	78	41.0	-	59	38.0	-	0.55
Heart disease	25	13.0	-	18	12.0	-	0.65
Kidney disease	166	88.0	-	121	79.0	-	0.02
AKI	135	81.0	-	98	81.0	-	0.94
CKD	31	19.0	-	23	19.0	-	0.94
**Drugs**	-	-	-	-	-	-	0.13
ACEi	47	25.0	-	32	21.0	-	-
ARB	5	3.0	-	0	-	-	-
Spironolactone	5	3.0	-	2	0.6	-	-
BB	1	0.5	-	0	-	-	-
Potassium supplements	1	0.5	-	1	0.6	-	-
Trimethoprim	0	-	-	5	3.0	-	-
ACEi plus BB	2	1.0	-	2	1.0	-	-
ACEi plus ARB	0	-	-	1	0.6	-	-
ACEi plus pironolactone	1	0.5	-	1	0.6	-	-
ARB plus spironolactone	0	-	-	1	0.6	-	-
**Laboratory characteristics**							
[K^+^] (mmol/L)	6.6	-	6.0–7.2	6.1	-	5.7–6.7	< 0.01
[K^+^] range (mmol/L)	-	-	-	-	-	-	-
5.5–5.9 mmol/L (mild)	44	23.0	-	68	44.0	-	< 0.01
6.0–6.9 mmol/L (moderate)	80	4.0	-	66	43.0	-	-
≥ 7.0 mmol/L (severe)	65	34.0	-	21	13.0	-	-
Total [Ca^++^] (mmol/L)	2.04	-	1.85–2.23	1.96	-	1.82–2.16	0.29
Ionised [Ca^++^] (mmol/L)	0.88	-	0.67–1.08	0.90	-	0.72–1.06	0.75
Total [HCO_3_-] (mmol/L)	15.4	-	10.2–22.5	19.3	-	13.0–26.0	< 0.01
Serum creatinine (µmol/L)	345	-	151–887	229	-	103–460	< 0.01
**In-hospital death**							
Died	56	30.0	-	38	25.0	-	0.29

Note: Laboratory characteristics ECG changes: pH, mean ± SD: Yes = 7.23 ± 0.16; No = 7.28 ± 0.11; *p*-value = < 0.01.

ECG, electrocardiogram; IQR, interquartile range; HIV, human immunodeficiency virus; AKI, acute kidney injury; CKD, chronic kidney disease; ACEi, angiotensin-converting enzyme inhibitor; ARB, angiotensin receptor blocker; BB, beta-blocker; [K^+^], serum potassium concentration; [Ca^++^], serum calcium concentration; [HCO_3_-], serum bicarbonate concentration.

Patients who had ECG changes had a higher median serum [K^+^] (6.6 mmol/L vs. 6.1 mmol/L, *p* < 0.01), lower serum pH (7.23 vs. 7.28, *p* < 0.01), lower serum bicarbonate concentration (15.4 mmol/L vs. 19.3 mmol/L, *p* < 0.01) and a higher serum creatinine concentration (345 µmol/L vs. 229 µmol/L, *p* < 0.01) (Table 3).

**TABLE 3:** Distribution of electrocardiographic abnormalities.

Type of ECG changes	ECG changes						*P*
	Yes (*N* = 189; 55%)			No (*N* = 155; 45%)			
	*n*	%	IQR (in ms)	*n*	%	IQR (in ms)
***P* waves**							
Median duration	112	-	98–128	100	-	92–106	**< 0.01**
Duration > 120 ms	58	31.0	-	-	-	-	-
Flat	12	6.0	-	-	-	-	-
Absent	28	15.0	-	-	-	-	-
**PR-interval**							
Median duration	164	-	143–191	142		128–154	**< 0.01**
Duration > 200 ms	32	17.0	-	-	-	-	-
1st degree AVB	25	13.0	-	-	-	-	-
2nd degree AVB	0	-	-	-	-	-	-
3rd degree AVB	2	1.0	-	-	-	-	-
**QRS complex**							
Median duration	96	-	84–106	86	-	78–96	< 0.01
Duration > 120 ms	27	14.0	-	-	-	-	-
LBBB	5	3.0	-	-	-	-	-
RBBB	8	4.0	-	-	-	-	-
Non-specific intraventricular conduction delay	7	3.7	-	-	-	-	-
Bifascicular block	1	0.5	-	-	-	-	-
Left anterior hemiblock	3	2.0	-	-	-	-	-
Pseudo-LBBB	1	0.5	-	-	-	-	-
Pseudo-RBBB	2	1.0	-	-	-	-	-
Sine waves	6	3.0	-	-	-	-	-
**QT interval**							
Corrected QT interval (Hodges)	431	-	408–459	412	-	399–426	**< 0.01**
Corrected QT interval (Hodges) for male	430	-	399–456	408	-	400–420	**< 0.01**
Corrected QT interval (Hodges) for females	435	-	414–460	416	-	399–428	**< 0.01**
QTc > 470 ms (female)	15/81	19.0	-	-	-	-	-
QTc > 450 ms (male)	32/108	30.0	-	-	-	-	-
***T* wave**							
Median *T* wave amplitude	7	-	4–10	6	-	4–8	0.05
Peaked *T* waves	84	45.0	-	-	-	-	-

ECG, electrocardiogram; IQR, interquartile range; AVB, atrioventricular block; LBBB, left bundle branch block; RBBB, right bundle branch block.

Patients who had hyperkalaemic ECG changes had increased duration of *p* waves (112 ms vs. 100 ms, *p* < 0.01), PR interval (164 ms vs. 142 ms, *p* < 0.01), QRS interval (96 ms vs. 86 ms, *p* < 0.01), higher corrected QT interval (413 ms vs. 412 ms, *p* < 0.01) as well as higher corrected QT interval by female sex (435 ms vs. 416 ms, *p* < 0.01) and by male sex (430 ms vs. 408 ms, *p* < 0.01) (Table 4).

**TABLE 4:** Multilinear regression for electrocardiogram changes associated with the [K^+^].

ECG alterations	*β* coefficient	SE	*t*	*P*	95% CI
*P* wave (ms)	−0.0056	0.0020	−2.81	< 0.01	−0.0095 to -0.0017
PR interval (ms)	0.0039	0.0011	3.66	< 0.01	0.0018 to 0.0060
QRS width (ms)	0.0076	0.0027	2.75	< 0.01	0.0022 to 0.0130
QTc (Hodges) (ms)	−0.0015	0.0015	−0.98	0.33	−0.0044 to 0.0015
*T* wave amplitude (mm)	0.0175	0.0113	1.55	0.12	−0.0047 to 0.0398
Heart rate (bpm)	0.0018	0.0019	0.94	0.35	−0.0019 to 0.0055

ECG, electrocardiogram; [K^+^], serum potassium concentration; SE, standard error; CI, confidence interval; bpm, beats per minute.

**FIGURE 2:**
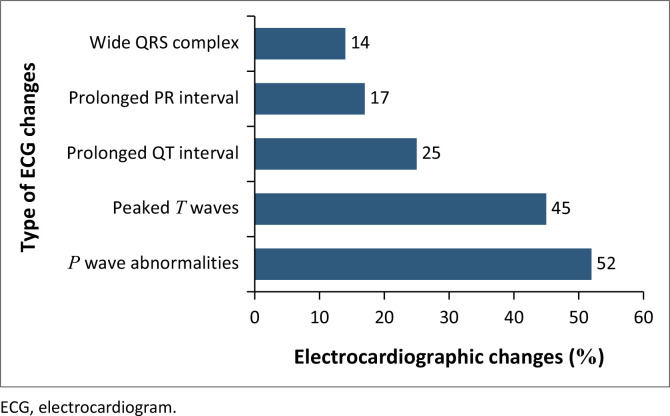
Frequency of electrocardiographic abnormalities.

The most common ECG features were *p*-wave abnormalities in 98 (52%) and peaked *T* waves in 84 (45%) (Figure 2). The distribution of the *p*-wave abnormalities was a duration > 120 msec in 58 (31%), flat *p* waves in 12 (6%) and absent *p* waves in 28 (15%) (Table 4).

**FIGURE 3:**
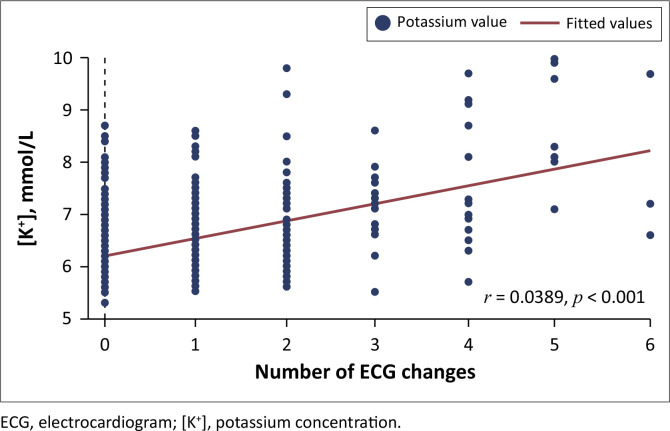
Correlation between the number of changes per electrocardiogram and the serum potassium concentration.

Using the Spearman correlation coefficient, ECG features associated with the [K^+^] were: PR duration (*r* = 0.171, *p* < 0.01), QRS duration (*r* = 0.181, *p* < 0.01) and heart rate (*r* = –0.125, *p* = 0.02); while the *T* wave amplitude (*r* = 0.065, *p* = 0.23), *p* wave duration (*r* = –0.042, *p* = 0.47), and the corrected QT interval (*r* = 0.071, *p* = 0.19) were not associated with the [K^+^]. Also, there was a weak-to-moderate correlation between the number of changes per ECG and the serum [K^+^] (*r* = 0.389, *p* < 0.01) (Figure 3). On multilinear regression, *p* wave, PR interval and QRS durations were associated with the [K^+^] (Table 4).

## Discussion

Among patients diagnosed with hyperkalaemia, we observed that at least half of them exhibited ECG alterations. Our findings align with those of other studies. For instance, a retrospective observational study reported ECG changes in 46% of patients,^10^ while another retrospective study indicated that 50% of patients displayed ECG abnormalities attributed to hyperkalaemia.^11^ In contrast, a prospective study found that three-quarters (75%) of hyperkalaemic patients showed ECG alterations.^12^ Several factors may contribute to these varying prevalence rates. These include discrepancies in study methodologies, such as retrospective versus prospective designs, as well as differences in patient demographics and comorbidities like diabetes mellitus and cardiovascular disease. Study settings also play a role, ranging from patients presenting to the emergency department to a selected group of dialysis patients. Furthermore, variations in the aetiology of hyperkalaemia, such as AKI versus chronic or end-stage kidney disease, along with distinct criteria for defining ECG changes during hyperkalaemia, could account for the disparities. Additionally, the distribution of mild, moderate or severe hyperkalaemia among patients may differ across studies. Another plausible explanation for the inconsistent prevalence rates is the inclusion of patients who may have experienced pseudohyperkalaemia.^13^ Therefore, it is crucial for clinicians to recognise that ECG changes during hyperkalaemia may have a wide prevalence range, which may limit its screening value.

To our knowledge, this study is the largest in sub-Saharan Africa exploring the relationship between hyperkalaemia and ECG alterations. The overall prevalence of ECG changes due to hyperkalaemia was only 55%. We found that the most frequently observed ECG alteration was abnormalities in *p* waves. This occurrence may have resulted from the varying sensitivity of myocardial tissue to the effects of hyperkalaemia. Research suggests that atrial tissue might be more susceptible compared to ventricular tissue.^14^ Other studies indicate a higher prevalence of peaked *T* waves.^4,11,15,16^ Due to the lack of a standardised definition for peaked *T* waves in hyperkalaemia, clinicians might tend to overinterpret this finding, considering it a well-known ECG indicator of hyperkalaemia.

We observed that patients exhibiting ECG changes were more commonly male, older in age, had kidney disease with higher serum creatinine concentrations and had more severe hyperkalaemia and lower serum pH or serum bicarbonate concentration. Similar findings were reported by another study.^17^ The rationale behind the male predominance remains unclear, whereas the more pronounced metabolic acidosis likely reflects the severity of underlying kidney dysfunction. Moreover, alongside reduced renal potassium elimination in patients with severe kidney dysfunction, metabolic acidosis may exacerbate hyperkalaemia by redistributing potassium from intracellular to the extracellular fluid compartment. The cardiac conduction system of older patients might be more susceptible to the effects of hyperkalaemia.^18^

We found poor correlations between all ECG changes with [K^+^] and a statistically significant weak-to-moderate correlation between the number of ECG changes and the severity of hyperkalaemia. This finding is consistent with those reported by others.^10,17,19^ Rafique et al. observed an increasing prevalence of any ECG abnormality as the potassium concentration rose from 5.5 mmol/L to > 7.0 mmol/L, while Varga et al. found that nearly half of the patients with hyperkalaemia exhibited ECG changes compared to only 24% of patients with normokalaemia.

This study has some strengths and limitations. This study had a large sample size. We could not identify any studies from Africa describing the prevalence of ECG changes during hyperkalaemia. Therefore, this study addresses this knowledge gap. Investigators were standardised by the utilisation of defined ECG criteria; however, the lack of universal definitions for ECG morphological changes associated with hyperkalaemia, especially regarding peaked *T* waves may affect our observed prevalence. On the other hand, this was a retrospective study; therefore, it is possible that several cases of hyperkalaemia were missed, and missing data at random could have introduced bias. The prevalence of ECG changes might have been affected by patients who received calcium salts for cardioprotection and other potassium-lowering therapies, as we also included those who had an ECG performed up to 3 h after documented hyperkalaemia. The diagnoses of AKI and CKD were documented based on patient medical charts. Therefore, we cannot guarantee the accuracy of these diagnoses. Even though we did not collect data on the prescription of digoxin, it is rarely used at our institution. Nonetheless, it may have confounded the interpretation of ECG changes.

## Conclusion

To our knowledge, this study is the largest in sub-Saharan Africa exploring the relationship between hyperkalaemia and ECG alterations. The overall prevalence of ECG changes due to hyperkalaemia was only 55%. Therefore, it is essential for clinicians to recognise that the ECG changes during hyperkalaemia may have limited screening value.
